# Understanding human arm stiffness modulation in overground pHRI: The roles of kinematics, perturbation, and trunk sway

**DOI:** 10.1371/journal.pone.0344748

**Published:** 2026-03-30

**Authors:** Mohsen Mohammadi Beirami, Sambad Regmi, Devin Burns, Yun Seong Song

**Affiliations:** 1 Department of Mechanical and Aerospace Engineering, Missouri University of Science and Technology, Rolla, Missouri, United States of America; 2 Department of Psychological Science, Missouri University of Science and Technology, Rolla, Missouri, United States of America; Polytechnic University of Marche: Universita Politecnica delle Marche, ITALY

## Abstract

This study examines human arm kinematics during overground physical human-robot interaction (pHRI). Previous work showed humans adjust arm stiffness with changing trajectory uncertainty, but the roles of arm kinematics and muscle activation remained unclear. Building on a preliminary study, we analyzed arm movements with more participants (10 individuals) to achieve more reliable findings and examined two potential influences that arose in the preliminary study: the robot’s perturbation effect (a brief hand push) and left-to-right trunk sway. Using a linear mixed-effects model, we evaluated the effects of participant, block, and trajectory condition on arm angles. Results showed minimal kinematic contribution to stiffness modulation, with inconsistent significance levels in the measured metrics. Perturbation presence also had no significant impact on voluntary posture, with the exception of the posture differences before and after the perturbation. Trunk sway was strongly correlated with elbow angle, with a mean correlation (*R*^2^) of 0.65 and a standard deviation of 0.24. Most variability arose from individual differences rather than experimental conditions. These findings might potentially allow for more flexible mechanical design in assistive and rehabilitation robots.

## Introduction

Effective motor communication is essential for safe and intuitive physical human-robot interaction (pHRI). While visual and auditory cues can provide valuable information about ongoing tasks—particularly in social HRI—the mechanical feedback inherent in the interaction itself offers crucial insights into the partner’s state [1]. Indeed, research suggests that humans rely on sensory information from physical interaction with their environment for better performance [1–6]. For example, people can skillfully maintain a stable grip on a mug in unsteady conditions [7] and differentiate between a friendly embrace and an unfriendly one [8]. Additionally, individuals in physical contact improve their own motor performance by considering their partner’s intended movement goal [9].

To develop robots capable of physical human-robot interaction and suitable control strategies, especially for rehabilitation purposes involving overground tasks, it is vital to understand human behavior and motor communication during these interactions [1,10–15]. Mechanical properties like force and displacement are central to studying these dynamics [1]. Among these properties, impedance—a measure linking forces to motion—is particularly valuable for analyzing physical interactions [16,17]. Our previous work [18] demonstrated the importance of mechanical impedance in pHRI. Experimental results from [5] showed that people adjust their arm stiffness depending on the predictability of a robotic partner’s movements; when participants were confident about the robot’s movement trajectory, they increased stiffness and required less haptic information. Conversely, when the robot’s future movements were uncertain, participants lowered stiffness to enhance sensitivity to small forces and detect movements more accurately. This finding aligns with research showing that low stiffness helps discriminate subtle forces through amplified proprioceptive feedback [1].

The ability to intentionally modulate arm stiffness during pHRI for effective motor communication raises questions about the mechanisms behind this adjustment [5]. Previous studies indicate that humans can modulate stiffness by activating muscles, adjusting arm posture, or using a combination of both strategies [16]. In overground pHRI tasks, which involve many degrees of freedom and fewer dynamic constraints, participants are not limited to a specific strategy or combination [5]. This flexibility suggests that the stiffness modulation strategy can be voluntarily selected for specific biomechanical reasons. Understanding how humans adjust stiffness could advance our knowledge of motor communication biomechanics and help the design of pHRI robots that are more intuitive for humans to interact with. Researchers in [19] initially examined the arm kinematics of participants during experiments in [5], revealing minimal kinematic influence on arm stiffness modulation in that study. In that preliminary study, a method was established to analyze arm kinematics during overground pHRI to examine its role in voluntary arm stiffness changes. The results showed inconsistent significance across metrics, suggesting no clear differences in arm kinematics between conditions. However, the study left open questions about factors that might influence arm kinematics in overground pHRI. For instance, the expectation of short mechanical perturbation—which is necessary to measure arm stiffness—may influence participants’ responses. Additionally, the large trunk sway that occurs during walking—a feature unique to overground tasks—may dominate arm kinematics modulation. One key question raised by [19] was whether arm kinematics differed before and after the voluntary response to the perturbation. It is well known that humans require time to respond to a stimulus; reactions to haptic stimuli typically occur at around 155 ms [20,21]. The overground experiment, with fewer constraints than traditional seated pHRI experiments [5], allowed participants more freedom in choosing their movement strategies. This leads to the hypothesis that the large rhythmic side-to-side trunk movements from overground walking tasks would affect arm kinematics.

In the present study, we address these gaps by: a) analyzing a larger cohort to obtain more reliable conclusions, b) examining the effect of external perturbations, and c) considering whole-body movement features such as the mediolateral trunk sway. This approach allows us to better understand the biomechanical strategies humans adopt during overground pHRI and clarifies the influence of arm kinematics on arm stiffness modulation.

## Materials and methods

In this study, we examine arm kinematics from the experiment reported in [5], building on the method initiated in [19], to address questions raised there about factors that may affect arm kinematics. Specifically, we investigate whether the arm stiffness modulation observed in [5] can be explained by arm kinematics, and we assess the potential influence of two factors identified in the preliminary work: the presence of a force perturbation (a brief robot-applied push) and left–to–right trunk sway during walking. To account for possible voluntary kinematic adjustments to imminent perturbations, we calculated and analyzed average elbow and shoulder angles. To explore the sway effect, we examined correlations between arm kinematics and trunk movement.

**Fig 1:**
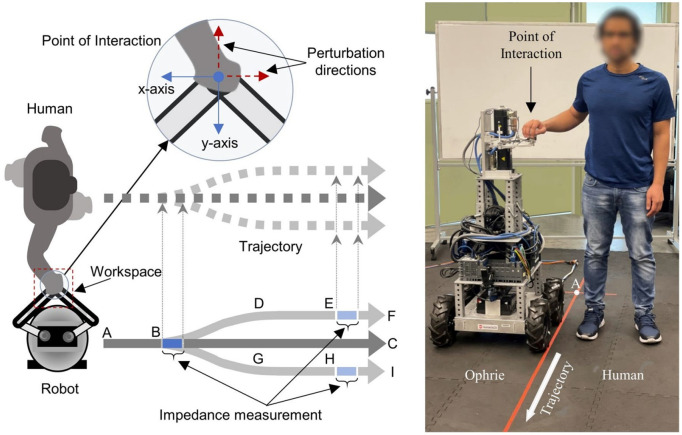
Experimental setup for overground physical human-robot interaction (pHRI) with OPHRIE guiding participants along one of three trajectories: straight (A-B-C) or turning (A-B-D-E-F, A-B-G-H-I). Figure adapted from [5].

In our previous work [5], we conducted an experiment to investigate how arm stiffness changes with different levels of uncertainty in a partner robot’s movement during overground human–robot interaction, as shown in Fig 1. The measured interaction force and the resulting hand movements were used to calculate the arm’s stiffness at those moments [5]. Arm stiffness was estimated through linear regression [10] using the interaction force (measured with a force sensor) and the handle movement (calculated from the manipulator’s kinematics). That experimental design compared stiffness at moments when participants were uncertain about the robot’s upcoming movement (point B) with moments when they were certain (points E or H). Results showed higher arm stiffness when participants were certain of the trajectory [5].

### Experimental protocol

**Fig 2:**
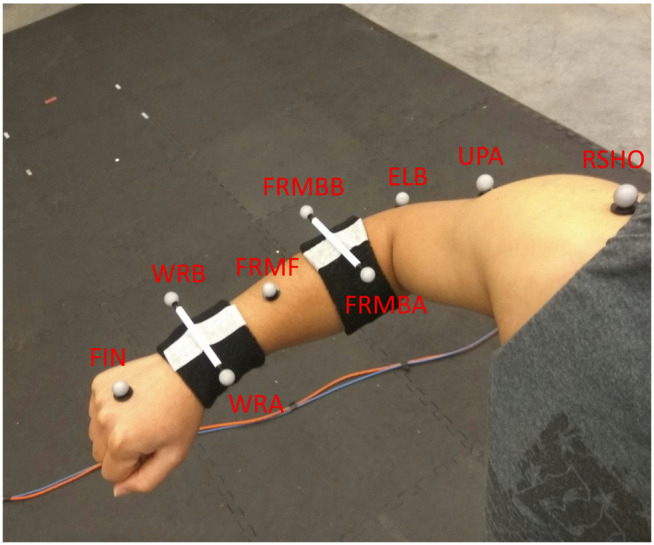
Reflective marker placement used to capture arm kinematics during the experiment [22].

In the experiment described in [5], our custom Over-Ground Physical Human–Robot Interaction Experiment (OPHRIE) robot guided blindfolded participants along one of three trajectories (one straight, two turning—left and right) while measuring stiffness at specific path points (Fig 1). Participants were instructed to stand upright next to OPHRIE and hold a doorknob-shaped handle with their right hand, ensuring secure contact with all fingers and the palm while avoiding isolated finger movements. The robot’s height was individually adjusted so that the handle was aligned with each participant’s elbow level. Participants were informed that once they were ready, the background stiffness controller would be activated. At that moment, they were asked to relax their arm, allowing the robotic arm to automatically position the handle at the center of its workspace—a 0.15 × 0.15 m² plane parallel to the ground. This procedure ensured a consistent and controlled starting arm position across all participants. After the experimenter gave a verbal cue, the robot initiated movement, and participants followed the robot with their eyes closed for the duration of the trial. Participants were instructed to walk naturally without controlling step length, step width, or cadence. In each trial, the path was chosen randomly, and the participant did not know which path the robot was taking until reaching point B, where the robot would either turn (B-D-E-F or B-G-H-I) or continue on the straight path (B-C). Point B represented the moment that participants were “uncertain” about the trajectory, and points E or H represented the moments when the participant was “certain” that OPHRIE would proceed directly toward either F or I, respectively. Force perturbations were applied at point B in straight trials or at E or H in turning trials, depending on turn direction, to measure arm dynamics. OPHRIE’s low-impedance manipulator applied a 3 N force for 800 ms and tracked handle motion at B, E, or H. Perturbations were directed either toward the participant (–y) or in the movement direction (–x). A background stiffness of 75 N/m held the handle near the workspace center; the stiffness controller was briefly disabled during the perturbation. OPHRIE maintained a linear velocity of 0.5 m/s on straight segments and an angular velocity of 0.45 rad/s during turns. Kinematic data were recorded at 200 Hz using Vicon Nexus and processed in ProCalc (Vicon Motion Systems, CO, USA). Marker trajectories and model outputs were filtered using Vicon Nexus 2.0’s built-in fourth-order zero-lag Butterworth filter with a 6 Hz cutoff frequency. Reflective markers (listed in Table 1 and shown in Fig 2) were placed on the right arm and upper body; nine markers were placed on the right arm and three additional markers were placed on the left shoulder (LSHO), the seventh cervical vertebra (C7), and the clavicular notch (CLV).

**Table 1:** List of markers and their anatomical locations.

Marker	Location
LSHO	Left shoulder
C7	7th cervical vertebra
CLV	Clavicle (upper end of the sternum)
RSHO	Right shoulder
UPA	Upper arm
ELB	Elbow joint
FRMBB	Forearm back B (pinky finger side)
FRMBA	Forearm back A (thumb finger side)
FRMF	Forearm front
WRB	Wrist B (pinky finger side)
WRA	Wrist A (thumb finger side)
FIN	Finger (on top of the hand)
LFB	Robot Base Plane (Left Front Base)
LBB	Robot Base Plane (Left Back Base)
RFB	Robot Base Plane (Right Front Base)
RBB	Robot Base Plane (Right Back Base)

The experiment employed a randomized complete block (RCB) design. Each of five blocks contained eight trials (four straight, four turning) assigned randomly. Ten participants completed the original study [5], yielding 400 trials in total. In [19], we analyzed the arm kinematics of the first four participants (160 trials) to establish the analysis method. In the present work, we build on that method to analyze the full dataset, analyzing 396 valid trials after excluding four unusable trials.

For the current analysis we used the raw data originally collected for [5], accessed for processing and analysis between 09/2024 and 12/2024 under Institutional Review Board approval. Ten healthy young adults (1 female, 1 left-handed; age 27.6 ± 2.41 years) who reported no prior history of neurological disorders participated in this study. Before participation, all subjects provided written informed consent. The Institutional Review Board of the University of Missouri System approved the research protocol, and procedures followed applicable guidelines and regulations.

### Data processing

**2D kinematic simplification (2D):** Arm motion in the experiment could be at most three degrees of freedom (DOF), and, due to the planar constraint of the handle workspace, the hand had two translational DOF within that workspace. Wrist rotations (e.g., radial–ulnar deviation) were minimal, and rotation about the axis formed by the wrist and shoulder joints was negligible. Maintaining a secure grip on the robot handle would relatively stiffen up the wrist joint and reduce movement. Consequently, the arm’s movement can be simplified to a 2 DOF representation, as noted in [5] and [19], modeled by elbow angle and shoulder angle.

**Fig 3:**
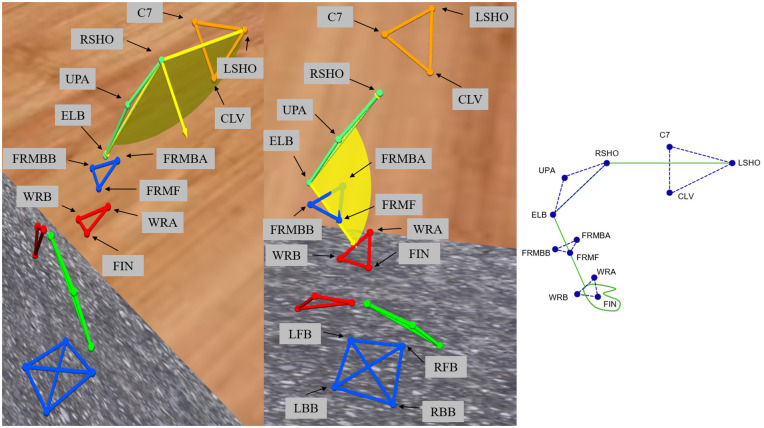
Elbow and shoulder angle representation in ProCalc software.

**Fig 4:**
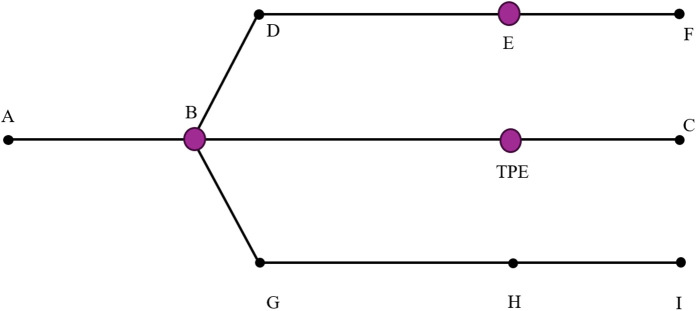
Illustration of the Turning Perturbation Equivalent (TPE). TPE in straight trials is positioned at the same distance from the starting point as points E and H in turning trials, but without applying a perturbation.

**Arm angle measurements:** Shoulder angle was calculated as the angle between vectors RSHO→LSHO and RSHO→ELB; elbow angle was calculated as the angle between ELB→RSHO and ELB→WRSTC, where WRSTC is the midpoint of markers WRA and WRB as represented in Fig 3. We used skin-mounted markers; markers used to extract arm angles were placed on bony landmarks of the arm. Multiple markers per segment were used, minimizing dependency on any single marker. Prior validation studies comparing skin-mounted to bone-fixed markers have shown that, when markers are carefully placed, joint angle errors are typically in the range of 1–3° for arm motion, supporting the accuracy of skin-marker-based measurements [23]. Given Vicon’s dynamic linear resolution of <0.1 mm and typical arm segment lengths (200 mm), the motion capture system provided acceptable accuracy for extracting arm kinematics. Because stiffness measurement requires applying a perturbation, direct stiffness estimates are not available at locations without a perturbation. To examine the effect of perturbation presence on kinematics, we introduced a Turning Perturbation Equivalent (TPE) point in straight trials. TPE is positioned at the same distance from the trial start as E/H (see Fig 4) but without a perturbation, providing a comparable moment for kinematic comparison between turning (E/H) and straight (TPE) trials. All trajectory segments used for comparison—including TPE and E/H—consisted of straight-line movements at matched velocities and similar body orientations, ensuring that TPE served as a fair control for perturbation-present turning trials. The robot’s velocity and orientation commands were identical for TPE and E/H points, ensuring that participants experienced the same experimental conditions. While minor trial-to-trial variations may occur, these commanded trajectories provide a valid basis for kinematic comparison between perturbation-present (E/H) and perturbation-absent (TPE) trials.

**Fig 5:**
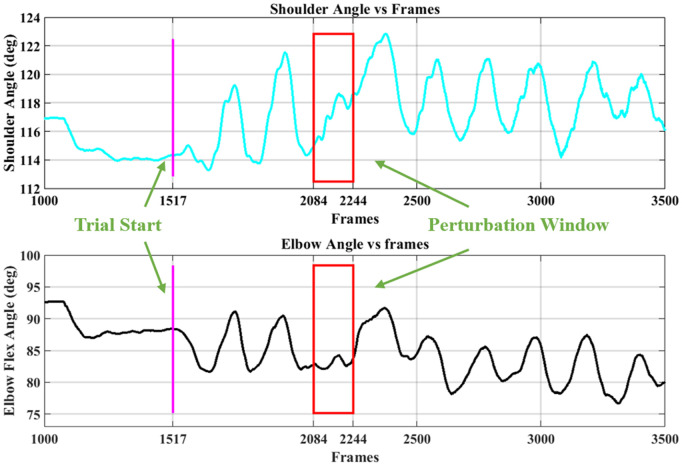
Example of shoulder and elbow angles during a straight trial. The perturbation window (160 frames, 800 ms) is shown in the red box and trial starting frame is marked with a vertical magenta line. Figure adapted from [19].

**Metrics:** We focused on joint angle adjustments at perturbation onset—Elbow Onset (EO) and Shoulder Onset (SO)—because these most directly relate to the stiffness measurements reported in [5]. However, large rhythmic side-to-side trunk movements during overground walking can introduce substantial variability in arm kinematics depending on the gait phase at perturbation onset. To minimize this influence, [19] computed average angles over a pre-perturbation window of approximately one stride (250 frames ≈ 1.25 s); these are Elbow Average Pre-perturbation (EAP) and Shoulder Average Pre-perturbation (SAP) in the current work. We also computed average angles over the 800 ms (160 frames) perturbation window to capture possible voluntary kinematic changes during perturbation; these are Elbow Average During-perturbation (EAD) and Shoulder Average During-perturbation (SAD). Fig 5 shows an example shoulder and elbow angle time series from a straight trial with a perturbation onset happening approximately 2.8 s after trial start.

### Statistical analysis

We tested the normality of the arm angle metrics and confirmed that both skewness and kurtosis magnitudes are less than 2. We also visually checked the normality of the data using R-studio’s qqnorm() function and did not see an indication of major deviation from normality. We compared:

- EO, EAP, EAD, SO, SAP, and SAD between point B (straight trials, uncertain) and E/H (turning trials, certain) to evaluate whether kinematics explain the stiffness differences reported in [5].
- EAP, EAD, SAP, SAD, and their differences (EAdiff=EAD−EAP, SAdiff=SAD−SAP) between E/H (turning, perturbation-present) and TPE (straight, perturbation-absent) to test the effect of perturbation presence on kinematic adjustments.

All comparisons used linear mixed-effects models to examine fixed effects of condition (straight (or TPE) vs turning), block (1–5), and random effects of participant. Although stiffness is inherently nonlinear with respect to continuous variables such as joint angle and muscle activation, the predictors in our analyses are primarily binary; therefore, linear mixed-effects models provide a straightforward and interpretable framework to assess whether effects differ significantly from zero. Statistical analyses were conducted in R (Posit Software, formerly RStudio, MA, USA) with α=0.05. Our data is available through the Harvard Dataverse (https://doi.org/10.7910/DVN/VMXN7A).

## Results

### Participant variability

**Fig 6:**
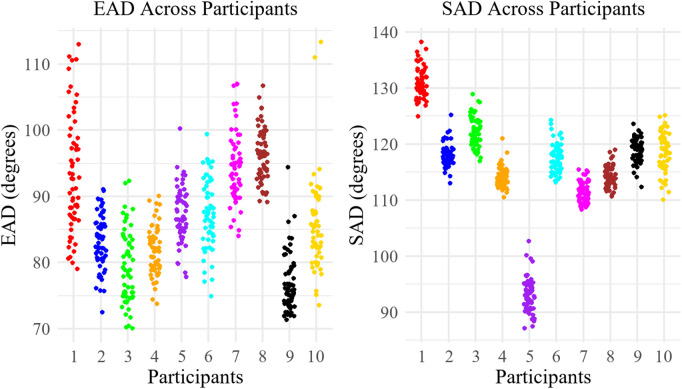
Scatter plots illustrating EAD and SAD values across individual participants.

Participant variability significantly influenced both elbow and shoulder angles, as shown in Table 2. Over 92% of the variation in shoulder angles, both onset and average measurements, was due to differences between participants. Similarly, for elbow angles, participants accounted for more than 60% of the total variance in both onset and average angles. To further illustrate these findings, Fig 6 presents scatter plots of EAD and SAD that visualize subject-level variations and highlight individual differences across participants.

**Table 2:** Variation in elbow and shoulder angles attributed to participant differences.

metric		participant	residual	participant/total
*Elbow*	EO	47.06°	30.56°	61%
	EAD	41.95°	28.37°	60%
	EAP	45.94°	26.89°	63%
*Shoulder*	SO	93.72°	7.56°	92%
	SAD	92.88°	5.88°	94%
	SAP	94.12°	6.11°	94%

### General trends in arm angles

**Fig 7:**
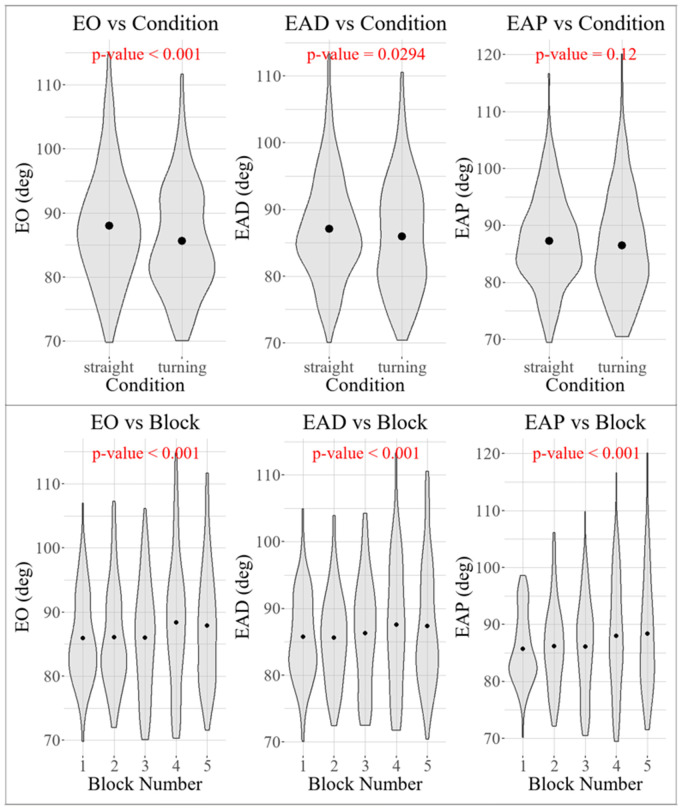
Violin plot comparing elbow angle in turning and straight trials and across the blocks, with the dot indicating the mean value.

Results from the mixed-effects model are summarized in Table 3. As shown in Fig 7, the elbow onset angle (EO) was significantly lower in turning trials—where the perturbation could be anticipated—than in straight trials (*p* < 0.001), with an average difference of 2.39°. This value closely matches the 2.37° difference reported in [19]. Block number had a significant effect on EO (*p* < 0.001), with EO increasing by 0.69° per block. A similar trend was observed for the average elbow angle before perturbation (EAP), which increased by 0.74° per block (*p* < 0.001); however, EAP did not differ significantly between turning and straight trials (*p* = 0.12). The average elbow angle during the perturbation window (EAD) also increased with block number (*p* < 0.001), showing a rise of 0.73° per block. EAD was significantly lower in turning trials than in straight trials, with a difference of 1.17° (*p* = 0.03).

**Table 3:** Mixed-effects model results for elbow and shoulder angles.

Metric		Estimate	Std Error	t value	Pr(> |*t*|)
**EO**	Interce	85.91°	2.26°	38.07	$1.97\times 10^{-12}$
	Block	0.69°	0.16°	4.28	$2.16\times 10^{-5}$
	Condition Turning	−2.39°	0.56°	−4.30	$2.01\times 10^{-5}$
**EAP**	Interce	84.97°	2.22°	38.25	$2.46\times 10^{-12}$
	Block	0.74°	0.15°	4.91	$1.19\times 10^{-6}$
	Condition Turning	−0.81°	0.52°	−1.55	0.12
**EAD**	Interce	84.83°	2.13°	39.75	$1.13\times 10^{-12}$
	Block	0.73°	0.15°	4.74	$2.63\times 10^{-6}$
	Condition Turning	−1.17°	0.54°	−2.18	0.0294
**SO**	Interce	116.51°	3.08°	37.86	$2.22\times 10^{-11}$
	Block	−0.13°	0.08°	−1.70	$9.10\times 10^{-2}$
	Condition Turning	0.16°	0.28°	0.58	$5.63\times 10^{-2}$
**SAP**	Interce	116.28°	3.08°	37.75	$2.44\times 10^{-11}$
	Block	−0.20°	0.07°	−2.81	0.005
	Condition Turning	1.00°	0.25°	4.02	$6.42\times 10^{-5}$
**SAD**	Interce	116.16°	3.06°	37.96	$2.33\times 10^{-11}$
	Block	−0.23°	0.07°	−3.21	0.001
	Condition Turning	0.51°	0.24°	2.10	0.036

**Fig 8:**
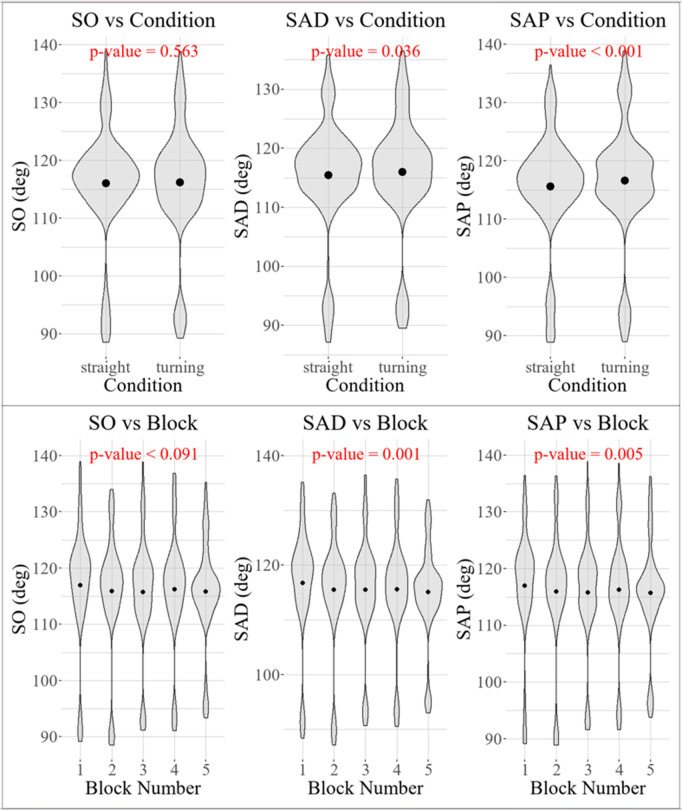
Violin plot comparing shoulder angle in turning and straight trials and across the blocks, with the dot indicating the mean value.

As represented in Fig 8, for the shoulder onset angle (SO), neither block number (*p* = 0.09) nor trial type (*p* = 0.56) had a significant effect. In contrast, the average shoulder angle before perturbation (SAP) decreased by 0.20° per block (*p* = 0.005) and was 1.00° higher in turning trials than in straight trials (*p* < 0.001), consistent with the 1.2° difference reported in [19]. The average shoulder angle during the perturbation window (SAD) decreased by 0.23° per block (*p* = 0.001). Turning trials also showed significantly higher SAD values than straight trials, with a difference of 0.51° (*p* = 0.036).

### Perturbation effect

**Fig 9:**
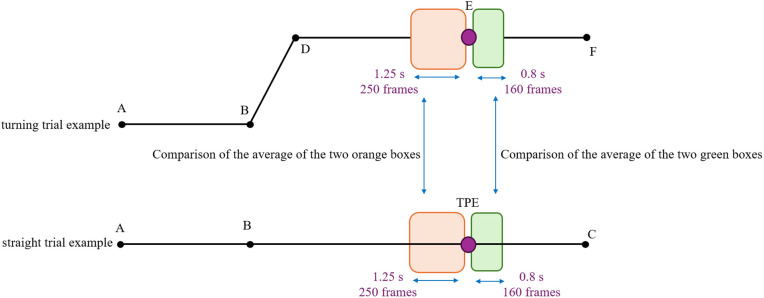
Visual representation of comparing average arm angles between the perturbation-present and perturbation-absent scenarios.

To check for the effect of the perturbation presence on arm kinematics, average elbow, and average shoulder angles before point TPE (average of 250 frames) were compared to average elbow and average shoulder angles before point E/H (EAP and SAP). Also, the average elbow and average shoulder angles after point TPE (average of 160 frames) were compared to average elbow and average shoulder angles after point E/H (EAD and SAD) as shown in Fig 9. Consistent significance levels were not achieved when comparing the perturbation-present and perturbation-absent scenarios (Table 4). Based on the results, it is not likely that the presence of perturbation affects the arm angle.

**Table 4:** Significance level of condition in the linear mixed effects model affecting different kinematic metrics (before E/H vs before TPE, after E/H vs after TPE).

metric	significance
**Elbow angle average before TPE and E/H (EAP)**	0.06
**Elbow angle average after TPE and E/H (EAD)**	0.12
**Shoulder angle average before TPE and E/H (SAP)**	** $1.35\times 10^{-6}$ **
**Shoulder angle average after TPE and E/H (SAD)**	0.45

**Fig 10:**
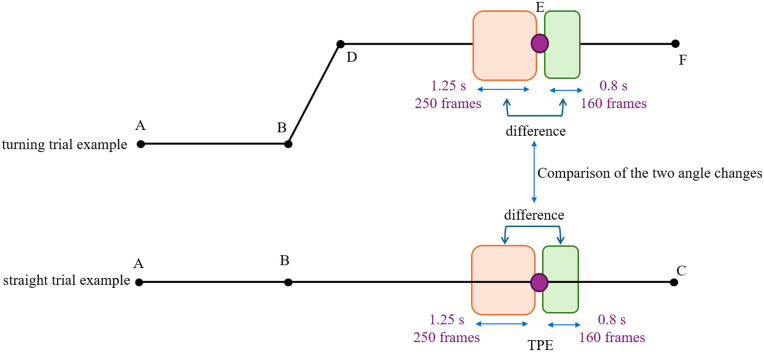
Visual representation of comparing the change in average arm angles between perturbation-present and perturbation-absent scenarios.

Although the effect of perturbation on arm angles was not conclusive, the change in arm angles depicted in Fig 10 between before and after (after minus before) point E/H (perturbation present) could still be significantly different from the change in arm angles between before and after point TPE (perturbation absent). To investigate these changes, the differences in arm angles before and after point TPE, as well as before and after points E/H, were calculated using Eq. (1) and Eq. (2). Note that EAdiff and SAdiff are calculated trial-by-trial, not based on block-level averages (for example, EAD in a trial minus the EAP in the same trial). A linear mixed effects model was then created, with conditions (TPE and turning) as the fixed effects and participants as random effects.


EAdiff=EAD−EAP (1)

SAdiff=SAD−SAP (2)

The mixed effects model results, shown in Table 5, indicate that there is a significant difference in arm angle change between before and after points E/H when compared to the changes between before and after TPE.

**Table 5:** Mixed effects model results for changes in average elbow (EAdiff) and shoulder (SAdiff) angles.

Metric		Estimate	Std Error	t value	Pr(> |*t*|)
**EAdiff**	Interce	1.22°	0.51°	2.36	0.02
	Block	0.01°	0.13°	0.11	0.92
	Condition Turning	−1.79°	0.38°	−4.68	$4\times 10^{-6}$
**SAdiff**	Interce	0.89°	0.23°	3.86	$2.03\times 10^{-4}$
	Block	−0.07°	0.06°	−1.17	0.24
	Condition Turning	−1.37°	0.17°	−8.24	$2.72\times 10^{-15}$

### Effect of trunk sway

**Fig 11:**
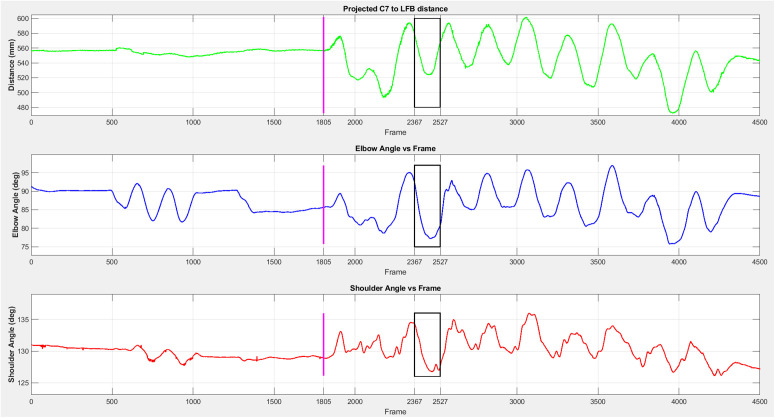
Elbow angle signal, shoulder angle signal, and $C_{7}\;projection-LFB\;distance$.

Due to the nature of the overground pHRI task during walking, body kinematics are heavily influenced by the cadence of walking [19]. This is evident in Fig 11 where both shoulder and elbow angles fluctuate considerably. To quantify the effect of the mediolateral (side-to-side) sway on arm kinematics, we first quantified the trunk sway using the *C*_7_ marker position. We measured the distance between the *C*_7_ marker’s projection on the robot base plane and the robot’s LFB marker, which represents the rhythmic proximity between the participant and the robot. To explore this relationship, we calculated the correlation (*R*^2^) between the $C_{7}\text{ projection}-\text{LFB }$ distance and both elbow and shoulder angle signals. Based on Table 6 there is a substantial correlation between arm angle signal and left-to-right trunk sway, where the elbow angle was more heavily correlated to the sway than the shoulder angle did. Table 6 represents the elbow angle signal, shoulder angle signal, and the ($C_{7}\text{ projection}-\text{LFB distance }$) signal in a straight trial for subject 1.

**Table 6:** Correlation between the $C_{7}\text{ projection}-\text{LFB distance }$ and arm angles.

Measure	Mean	Standard Deviation
R² (Elbow Angle, D*)	0.65	0.24
R² (Shoulder Angle, D)	0.4	0.25

*D: $C_{7}\text{ projection}-\text{LFB distance }$.

## Discussion

Our findings align with those reported in [19] and also help address the questions raised. Similar to [19], arm kinematics appear to play a minimal role in arm stiffness modulation during over-ground human-robot interaction tasks. One notable difference from [19] is that the block numbers affect the average shoulder angles (SAD and SAP). However, the block-to-block effect was small (0.23° decrease). Considering that an experiment with each participant took 2–2.5 hours, this is a very slow change in the shoulder angle and the effect size may not become relevant unless the experiments lasted considerably longer.

The mixed-effects model showed that most variability originated from individual differences. While factors such as sex, arm length, height, or weight could contribute to this variation, they were not controlled in this study, as the focus was on capturing natural human variability representative of a broad user population. Since interactive robots are intended for use by diverse individuals, accounting for random demographic factors was outside the scope of this work.

This study focused on arm kinematics during overground physical human-robot interaction. Since this was the first pHRI study using a robot such as OPHRIE – an overground robot with a back-drivable manipulator arm with occasional force perturbation, no prior data was available to expect specific arm kinematic behavior in this experiment protocol. One key observation of this study is that arm stiffness modulation is unlikely to have been caused by arm kinematics. In our previous study [5], we observed differences in arm stiffness modulation between Point E/H (certain) and Point B (uncertain). However, a comparison of arm kinematics at these points suggests that humans are unlikely to rely on arm kinematics to modulate stiffness. This suggests that arm muscle activation may be the main cause of arm stiffness changes in different conditions. To better test this, future research could consider incorporating electromyography (EMG) for a more comprehensive understanding of arm stiffness modulation [24,25]. A similar perspective could be explored for lower-limb applications and the stiffness modulation due to ground reaction forces [26]. Additionally, exploring other variables, such as variations in walking speed, or the effect of handedness may provide further insights.

Our findings indicate that arm kinematics have minimal influence on stiffness modulation during overground pHRI. This has several practical implications for rehabilitation and robotic device design. First, it reduces the need for complex motion capture systems to track human arm movements in future experiments or clinical devices, simplifying setup and lowering associated costs. Second, the minimal kinematic variation provides greater mechanical flexibility in designing robotic devices or rehabilitation systems, as there are fewer constraints on how and where the user’s arm must be positioned. These insights can guide therapy planning, and the selection of sensors, allowing developers to focus on essential factors that influence arm stiffness modulation rather than precise kinematic tracking.

Comparing the perturbation-present points (E/H) with perturbations-absent points (TPE), the difference in average angles was insignificant. This demonstrates the participant’s knowledge of the presence or absence of perturbation did not affect how they adjust their arm kinematics. Future studies that compare arm kinematics in trials with and without force perturbations may consider this in the expected arm kinematics data. Nonetheless, between two conditions that both have force perturbations (such as in this study), this information may not be relevant.

The significance shown in the EAdiff and SAdiff metrics show that the amount of arm kinematics changes are different with or without perturbation. This is somewhat expected because external force from perturbation may contribute to the displacement of the arm.

Also, while the average angle measures may turn out to be not significantly different between E/H and TPE, their subtraction could show significance, especially since the average angle measures still show weak trends of differences, except for SAD.

The dominance of lateral sway on arm kinematics was highly noticeable, especially at the elbow angle. This suggests that in the arm configuration that we used, elbow angles are more effectively used to offset the effect of sway, as the elbow angle correlation to the sway was higher than for the shoulder angle. One possible explanation is that the shoulder joint may be intrinsically stiffer than the elbow joint due to the number and size of muscles across the shoulder joint. Also, since the elbow joint is more distal than the shoulder joint, it is affected more by the movement of the base of the kinematic chain of the body (trunk-shoulder-elbow). [3] and [27] explain that physical touch could improve stability while standing and walking. In [3] it is mentioned that higher interaction torques result in higher reductions in sway. Observing the strong correlation between elbow angle and body sway in our research, future physically interactive robots could potentially use elbow angle signals to assess the magnitude of interaction force or torque to improve the stability of the interaction. Furthermore, this relationship raises intriguing possibilities, such as whether the magnitude of lateral sway is influenced by the interaction with a partner, whether easier pHRI tasks might lead to increased sway, or whether factors like trust between partners could modulate sway dynamics.

In [28] the participants self-selected distinctively different arm poses in response to the direction of environmental instabilities. The endpoint (at the hand) stiffness of the arm was modulated by changing the arm poses. On the other hand, in this experiment, we observed only minimal changes in the arm kinematics between conditions (certain vs uncertain) that resulted in the arm stiffness change. This difference may be due to the nature of the task. In [28] the physical interaction was a static, standing task, whereas the task in this work was a dynamic, overground task. [28] investigated a stabilization task with stochastic perturbation, whereas, in this study, the task was a motor communication task with temporal perturbation. Based on these observations, the human arm kinematic movement strategy seems to differ between the given pHRI tasks. For example, while the participants in both studies ([28]) and this study) were allowed to select their preferred arm poses, participants in the overground pHRI studies may feel less inclined to drastically vary the arm poses because this was not part of the instructions given to them. The nature of the task (which resembles walking with another human) may have disposed them to a similar preferred arm pose, even when they were allowed to choose their own poses.

## Limitations and future work

While this study provides new insights into arm kinematics during overground human–robot interaction, certain aspects warrant further exploration. Muscle activity data were not included in the current setup, and only upper-limb markers were analyzed. Incorporating electromyography (EMG) and additional body markers in future experiments would enable a more comprehensive understanding of how muscle activation and whole-body dynamics contribute to arm stiffness modulation. Skin-mounted markers provide a non-invasive approach for in vivo measurements; they are still subject to soft tissue artifact, and thus, absolute joint angles may differ slightly from bone-fixed measurements. Nevertheless, careful marker placement on bony landmarks and use of multiple markers per segment minimize these errors, and relative differences reported here remain robust. Moreover, extending the walking path to include multiple gait cycles could offer deeper insights into the relationship between gait phase and arm kinematics.

## Conclusion

This study builds on previous work exploring the role of arm stiffness modulation during over-ground physical human-robot interaction (pHRI), specifically investigating the contribution of arm kinematics. Our findings reinforce earlier results, showing that arm kinematics have a minimal influence on arm stiffness modulation in this task. The use of a linear mixed-effects model confirmed that participant variability, rather than arm kinematics, was the primary source of variation in arm angles. Future research may incorporate EMG data as well as explore other factors that may affect arm stiffness modulation, such as varying walking speeds and data collection from the left arm, to gain a better understanding of the mechanisms behind arm stiffness modulation.

## References

[1] Rashid F, Burns D, Song YS. Sensing small interaction forces through proprioception. Sci Rep. 2021;11(1):21829. 34750408 10.1038/s41598-021-01112-w PMC8575958

[2] Holmes GL, Bonnett KM, Costa A, Burns D, Song YS. Guiding a Human Follower with Interaction Forces: Implications on Physical Human-Robot Interaction. In: 2022 9th IEEE RAS/EMBS International Conference for Biomedical Robotics and Biomechatronics (BioRob). IEEE; 2022. p. 1–6.

[3] Wu M, Drnach L, Bong SM, Song YS, Ting LH. Human-human hand interactions aid balance during walking by haptic communication. Frontiers in Robotics and AI. 2021;8:735575. 34805289 10.3389/frobt.2021.735575 PMC8599825

[4] Rashid F, Burns D, Song YS. Factors affecting the sensitivity to small interaction forces in humans. In: 2021 43rd Annual International Conference of the IEEE Engineering in Medicine & Biology Society (EMBC). IEEE; 2021. p. 6066–9. 10.1109/EMBC46164.2021.9629751 34892500

[5] Regmi S, Burns D, Song YS. Humans modulate arm stiffness to facilitate motor communication during overground physical human-robot interaction. Scientific Reports. 2022;12(1):18767. 36335247 10.1038/s41598-022-23496-z PMC9637182

[6] Saini A, Burns D, Emmett D, Song YS. Trunk velocity-dependent Light Touch reduces postural sway during standing. PLOS ONE. 2019;14(11):1–12. doi: 10.1371/journal.pone.0224943 PMC6837461 31697773

[7] Marsden CD, Merton PA, Morton HB. Human postural responses. Brain. 1981;104(3):513–34. 7272713 10.1093/brain/104.3.513

[8] Block AE, Christen S, Gassert R, Hilliges O, Kuchenbecker KJ. The six hug commandments: Design and evaluation of a human-sized hugging robot with visual and haptic perception. In: Proceedings of the 2021 ACM/IEEE international conference on human-robot interaction; 2021. p. 380–8.

[9] Takagi A, Ganesh G, Yoshioka T, Kawato M, Burdet E. Physically interacting individuals estimate the partner’s goal to enhance their movements. Nature Human Behaviour. 2017;1(3):0054.

[10] Regmi S, Burns D, Song YS. A robot for overground physical human-robot interaction experiments. Plos one. 2022;17(11):e0276980. 10.1371/journal.pone.0276980 PMC9648723 36355780

[11] Zendehdel N, Zadeh KG, Chen H, Song YS, et al. Hands-Free UAV Control: Real-Time Eye Movement Detection Using EOG and LSTM Networks. IEEE Access. 2025.

[12] Ghorbani Zadeh K, Zendehdel N, Holmes GL Jr, Bonnett KM, Zendehdel N, Costa A, et al. Comparison of CNN and LSTM Networks on Human Intention Prediction in Physical Human–Robot Interactions. In: 2025 IEEE 21st International Conference on Automation Science and Engineering. IEEE; 2025. p. 1–6.

[13] Tien H, Still R, Ghorbani Zadeh K, Mohammadi Beirami M, Burns D, Song YS. Development of a Force Perturbation Handle for Physical Interaction Research in Humans. ASME Journal of Biomechanical Engineering, under review. 2025;. 10.1115/1.4071214 41746300

[14] Soleimani E, Sedigh AK, Nikoofard A. Data-Driven Reinforcement Learning-Based Forgetting Factor Iterative Learning Control. IEEE Transactions on Automation Science and Engineering. 2025;22:12245–56. doi: 10.1109/TASE.2025.3540699

[15] Zadeh KG, Song YS. ESO-based Adaptive Control of Haptic Devices for Transparency Improvement and Human Active Force Estimation. techrxiv pre-print 2025,10.36227/techrxiv.176300517.79706882/v1

[16] Mussa-Ivaldi FA, Hogan N, Bizzi E. Neural, mechanical, and geometric factors subserving arm posture in humans. Journal of neuroscience. 1985;5(10):2732–43. 4045550 10.1523/JNEUROSCI.05-10-02732.1985 PMC6565149

[17] Gomi H, Kawato M. Human arm stiffness and equilibrium-point trajectory during multi-joint movement. Biological cybernetics. 1997;76(3):163–71. 9151414 10.1007/s004220050329

[18] Regmi S, Song YS. Design methodology for robotic manipulator for overground physical interaction tasks. Journal of Mechanisms and Robotics. 2020;12(4):041002.

[19] Beirami MM, Regmi S, Burns D, Song YS. Exploring Kinematics Contribution to the Arm Stiffness Modulation During Overground Physical Human-Robot Interaction. In: 2024 46th Annual International Conference of the IEEE Engineering in Medicine and Biology Society (EMBC). IEEE; 2024. p. 1–5. 10.1109/EMBC53108.2024.10781817 40039937

[20] Chapwouo Tchakouté LD, Tremblay L, Menelas BAJ. Response time to a vibrotactile stimulus presented on the foot at rest and during walking on different surfaces. Sensors. 2018;18(7):2088. 29966251 10.3390/s18072088 PMC6069424

[21] Kosinski RJ. A literature review on reaction time. Clemson University. 2008;10(1):337–44.

[22] Regmi S. Development of an interactive robot for overground physical human-robot interaction. Missouri University of Science and Technology; 2022.

[23] Naaim A, Moissenet F, Duprey S, Begon M, Chèze L. Effect of various upper limb multibody models on soft tissue artefact correction: A case study. Journal of Biomechanics. 2017;62:102–9. doi: 10.1016/j.jbiomech.2017.01.031 28274475

[24] Maintained SFESD. A Myokinetic Arm Model for Estimating Joint Torque. J Neurophysiol. 2009;101:387–401. 19005007 10.1152/jn.00584.2007

[25] Osu R, Gomi H. Multijoint muscle regulation mechanisms examined by measured human arm stiffness and EMG signals. Journal of neurophysiology. 1999;81(4):1458–68. 10200182 10.1152/jn.1999.81.4.1458

[26] Mengarelli A, Tigrini A, Scattolini M, Mobarak R, Burattini L, Fioretti S, et al. Myoelectric-Based Estimation of Vertical Ground Reaction Force During Unconstrained Walking by a Stacked One-Dimensional Convolutional Long Short-Term Memory Model. Sensors. 2024;24(23). doi: 10.3390/s24237768 PMC11645049 39686306

[27] Reynolds RF, Osler CJ. Mechanisms of interpersonal sway synchrony and stability. Journal of the Royal Society Interface. 2014;11(101):20140751. 25339686 10.1098/rsif.2014.0751 PMC4223902

[28] Trumbower RD, Krutky MA, Yang BS, Perreault EJ. Use of self-selected postures to regulate multi-joint stiffness during unconstrained tasks. PloS one. 2009;4(5):e5411. 10.1371/journal.pone.0005411 PMC2671603 19412540

